# A randomised feasibility trial of an employer-based intervention for enhancing successful return to work of cancer survivors (MiLES intervention)

**DOI:** 10.1186/s12889-021-11357-9

**Published:** 2021-07-21

**Authors:** M. A. Greidanus, A. E. de Rijk, A. G. E. M. de Boer, M. E. M. M. Bos, P. W. Plaisier, R. M. Smeenk, M. H. W. Frings-Dresen, S. J. Tamminga

**Affiliations:** 1 Department Public and Occupational Health/ Coronel Institute of Occupational Health, Amsterdam Public Health research institute, Amsterdam UMC, University of Amsterdam, Meibergdreef 9, Amsterdam, The Netherlands; 2 Department of Social Medicine, Care and Public Health Research Institute (CAPHRI), Faculty of Health, Medicine and Life Sciences, Maastricht University, Duboisdomein 30, Maastricht, The Netherlands; 3 Department of Medical Oncology, Erasmus Medical Center, Doctor Molewaterplein 40, Rotterdam, The Netherlands; 4 Department of Surgery, Albert Schweitzer Hospital, Albert Schweitzerplaats 25, Dordrecht, The Netherlands

**Keywords:** Feasibility studies, Employment, Neoplasms, Employer, Internet-based intervention, Manager, Pilot randomised controlled trial, Sick leave, Supervisor, Occupational health services

## Abstract

**Background:**

Employers express a need for support during sickness absence and return to work (RTW) of cancer survivors. Therefore, a web-based intervention (MiLES) targeted at employers with the objective of enhancing cancer survivors’ successful RTW has been developed. This study aimed to assess feasibility of a future definitive randomised controlled trial (RCT) on the effectiveness of the MiLES intervention. Also preliminary results on the effectiveness of the MiLES intervention were obtained.

**Methods:**

A randomised feasibility trial of 6 months was undertaken with cancer survivors aged 18–63 years, diagnosed with cancer < 2 years earlier, currently in paid employment, and sick-listed < 1 year. Participants were randomised to an intervention group, with their employer receiving the MiLES intervention, or to a waiting-list control group (2:1). Feasibility of a future definitive RCT was determined on the basis of predefined criteria related to method and protocol-related uncertainties (e.g. reach, retention, appropriateness). The primary effect measure (i.e. successful RTW) and secondary effect measures (e.g. quality of working life) were assessed at baseline and 3 and 6 months thereafter.

**Results:**

Thirty-five cancer survivors were included via medical specialists (4% of the initially invited group) and open invitations, and thereafter randomised to the intervention (*n* = 24) or control group (*n* = 11). Most participants were female (97%) with breast cancer (80%) and a permanent employment contract (94%). All predefined criteria for feasibility of a future definitive RCT were achieved, except that concerning the study’s reach (90 participants). After 6 months, 92% of the intervention group and 100% of the control group returned to work (RR: 0.92, 95% CI: 0.81–1.03); no difference were found with regard to secondary effect measures.

**Conclusions:**

With the current design a future definitive RCT on the effectiveness of the MiLES intervention on successful RTW of cancer survivors is not feasible, since recruitment of survivors fell short of the predefined minimum for feasibility. There was selection bias towards survivors at low risk of adverse work outcomes, which reduced generalisability of the outcomes. An alternative study design is needed to study effectiveness of the MiLES intervention.

**Trial registration:**

The study has been registered in the Dutch Trial Register (NL6758/NTR7627).

**Supplementary Information:**

The online version contains supplementary material available at 10.1186/s12889-021-11357-9.

## Background

Enhancing work participation of cancer survivors has gained more attention in the past few decades [1, 2]. This has been necessary, since almost half of persons diagnosed with cancer are of working age, and survival rates have continued to increase [3, 4]. Work is part of cancer survivors’ identity, and contributes to their well-being [5, 6]. Work can also be a well-appreciated distraction and provide an opportunity to stay socially active during treatments, to regain a sense of normality, and to earn a living [6]. Despite the attention paid to enhancing cancer survivors’ work participation, a significant proportion still have to deal with short and long-term adverse work outcomes due to their cancer, such as unemployment [7–10].

So far, most work-related interventions have focused on the cancer survivors themselves; for example, by means of education, counselling, or physical training [11, 12]. These interventions have shown inconclusive results on survivors’ work outcomes [11, 12]. This might argue in favour of shifting the focus to other stakeholders, such as occupational physicians and employers. Adequate support from the workplace is regarded as vital, and employer support has been found to be key in facilitating cancer survivors’ return to work [13]. By “employer”, we here mean the specific person who represents the organisation that employs the cancer survivor, supporting them during their sickness absence and RTW; for example, the supervisor, line-manager or human resources manager (HR manager). Employers themselves point out that providing RTW support to cancer survivors is challenging, at best [14–17]. Employers experience complex communication and decision-making dilemmas; for example, because survivor and organisational interests may contradict [16, 17]. Numerous studies have therefore emphasised the need for interventions targeted at employers, to improve their support for cancer survivors [11, 15–21]. As such, cancer survivors’ work outcomes may be enhanced via the employer.

We have developed such an employer-based intervention, named MiLES (an abbreviation of “the Missing Link: optimising return to work of Employees diagnosed with cancer, by Supporting employers”) [22]. This includes information, videos, and checklists to support employers in providing adequate RTW guidance when one of their employees is diagnosed with cancer. As a “one-sized-fits-all” approach will not meet the needs of every employer or survivor [23, 24], the MiLES intervention differentiates between 4 RTW phases (i.e. disclosure of cancer to the employer, treatment of the cancer survivor, RTW planning, and actual RTW). In addition, various experience types of cancer survivor are distinguished [25] and the content of the intervention is tailored to these experience types [22]. By providing employers with support so as to improve their RTW guidance to cancer survivors, the MiLES intervention aims to enhance successful RTW of cancer survivors [22].

In a previous study, we have assessed the use and perceived usefulness of the MiLES intervention among employers [26]. We found that most employers (*n* = 22; 82%) used the intervention, typically 2–3 times over a period of 6 weeks, and on average for 26 min per visit [26]. Employers perceived the intervention to be useful (i.e. perceived usefulness was scored 7.6 out of 10 points), and all participated employers would recommend the intervention to colleagues [26]. These results clearly show that, from the employer perspective, the MiLES intervention is a useful tool that is suitable to employers’ daily practice. The question of whether the MiLES intervention is actually effective in enhancing the successful RTW of cancer survivors, is unanswered. Knowledge on its effectiveness on cancer survivors’ work outcomes is urgently needed, so that cancer survivors and their employers can benefit from the MiLES intervention, when effectiveness has been proven.

Tools that intervene at the intersection between employer and cancer survivor are scarce [27, 28], and studies to determine the effectiveness of such tools on survivors’ work outcomes are, to the best of our knowledge, absent in scientific literature [19, 27, 28]. Various aspects complicate studying the effectiveness of an employer-based intervention on work outcomes of cancer survivors. For example, outcomes are measured in a different population (i.e. cancer survivors) than the population whose behaviour is intended to be changed by means of the intervention (i.e. employers). Combining these 2 layers in 1 study is impeded by ethical concerns and privacy regulations [29]. Including employer-employed survivor pairs is not allowed as it breaches privacy regulations concerning the exchange of health-related information between these 2 parties [29]. As a consequence, the measurement of variables by either side (e.g. work outcomes of the cancer survivors and their respective employers’ user data concerning the intervention) is also not possible, obviating the conduct of per-protocol analyses. Besides such complicating aspects, it is also uncertain whether cancer survivors are willing to participate in a study on the effectiveness of an intervention targeted at their employer. Taking these complicating aspects and uncertainties into consideration, it is essential to conduct a feasibility trial before conducting a future definitive trial on the effectiveness of the MiLES intervention on successful RTW of cancer survivors [30, 31]. For this, a so called “randomised feasibility study” is appropriate, since this type of feasibility study reflects the future definitive randomised controlled trial (RCT) on most parts, including randomisation [32]. By running the “smaller version of the future definitive RCT” a randomised feasibility trial can thus uncover obstacles for such a future definitive RCT, and thereby increase the probability that it will be successful. Or it can prevent a costly but unfeasible study being carried out [30, 33, 34].

The aim of this randomised feasibility trial was to assess feasibility of a future definitive RCT on the effectiveness of the MiLES intervention in terms of recruitment, reach, and acceptability of the study protocol. Secondary aims were: 1) to obtain preliminary results on the effectiveness of the MiLES intervention on successful RTW of cancer survivors, and 2) to determine the sample size needed in a future definitive RCT on the effectiveness of the MiLES intervention.

## Methods

The CONSORT 2010 extension for randomised pilot and feasibility trials was used to structure the reporting of this study [34]. This extension was developed based on the standard CONSORT checklist for RCTs, with some items being removed and some items specifically related to feasibility trials being supplemented, such as items on the reporting of prespecified criteria used to judge whether or not to proceed with a future definitive RCT [35]. The completed CONSORT checklist (retrieved from www.consort-statement.org) can be found in Additional file 1. The study has been registered in the Dutch Trial Register (NTR) (www.trialregister.nl; registration numbers NL6758 and NTR7627, dd. 30/10/2018). The objectives, development, and design of the MiLES intervention [22], as well as the study design [36] have already been described in detail elsewhere. The study protocol can also be found in Additional file 2. No significant changes were made with regard to the published design.

### Trial design

The study was conducted as a non-blinded randomised feasibility trial with a follow-up of 6 months. Eligible employed cancer survivors were randomised to an intervention group, in which cancer survivors were encouraged to inform their employer about the MiLES intervention, or a waiting-list control group, with the employer of the participating survivor not receiving the intervention for a period of 6 months. Participating cancer survivors filled in an online questionnaire at baseline, and at 3 and 6 months into follow-up.

### Participants

Based on feasibility-study size estimations [31, 33], we aimed to include 90 cancer survivors who met the following inclusion criteria: between 18 and 63 years of age; diagnosed with cancer < 2 years earlier; in paid employment under a temporary (> 6 months remaining) or permanent contract on a full-time, part-time, or flexible basis; currently fully or partially sick-listed for < 1 year; able to complete 3 questionnaires in the following 6 months (as assessed by their medical specialist on the basis of their current health and expectations about their health in the future); and able to understand, speak, and read Dutch sufficiently to fill out the questionnaires. We excluded cancer survivors who had not informed their employer about their diagnosis of cancer, in order to not to put them under unintended pressure to make that disclosure through the procedures of this study.

Potential eligible cancer survivors were recruited via medical specialists at 2 hospitals in the Netherlands. Cancer survivors received an invitation letter, information sheet, approach form, and return envelope, and were asked to indicate on the approach form whether they met the inclusion criteria and whether or not they were interested in participating. At 1 hospital, postal reminders were sent out 3 weeks after the invitation to those survivors who had not yet returned the approach form. In the event that a survivor indicated that they were interested in participating, MG or the research assistant contacted them to explain the study and to check their individual eligibility. Survivors were included after signing a digital informed consent form.

In addition to that outlined above, which has been described in the published design [36], we employed 3 additional recruitment strategies: open invitations distributed at drop-in centres for cancer survivors; a link on social media (Facebook and LinkedIn); and open invitations and a link in newsletters sent by Dutch cancer-related websites, such as the online platform www.kanker.nl. In response to all these recruitment strategies, cancer survivors who were eligible and interested in participating could sent a postal or digital approach form to the research team, after which MG or the research assistant contacted the respondent by telephone to check the their eligibility and, if they were indeed found to qualify, to include them in the study.

### Interventions

#### The MiLES intervention targeted at the participant’s employer

The MiLES intervention was developed using the comprehensive and systematic Intervention Mapping approach [22, 37], and consists of an online toolbox targeted at the participant’s employer. An extensive overview of the development, objectives and various components of this toolbox has been published elsewhere [22]. In short, a needs assessment was developed with input from, among others, employer interviews (*N* = 30) [16], a systematic review [23], and a Delphi study [24]. The toolbox targets the most important employer actions for successful RTW selected by employers and cancer survivors [22, 24]. The Resource Dependence Institutional Cooperation (RDIC) model was used [23, 38], which assumes that whether an employer performs a specific employer action properly is determined by their willingness and their ability to support. It was assumed that support from the employer will be adequate if the employer is willing and able to perform the most important employer actions and to tailor these actions based on the preferences and needs of their specific cancer survivor [22]. Additionally, the trans-theoretical model of change was used as a theoretical framework for making changes, and several methodologies were employed to induce the desired behaviour change, for example modelling, goal setting, tailoring, active learning and scenario-based risk evaluation [22]. The toolbox was develop as a web-based intervention with succinct information, interactive communication videos, conversation checklists, and links to reliable external sources [22]. The content of the toolbox is tailored per RTW phase and per cancer survivor experience type. For the latter, 3 predefined types are distinguished [25]: 1) an *emotional cancer survivor*, in which intense emotions such as sadness and anger can alternate quickly; 2) a *cancer survivor who wants little attention for their health situation*, and wants to be involved in work for as long as possible and return to work as quickly as possible; and 3) a *cancer survivor who starts looking differently at work and life*, and gives other priorities due to their illness. Employers who used the toolbox have indicated that it increased both their ability and their willingness to support cancer survivors (94 and 66% of the participated employers, respectively) [26]. The toolbox is accessible via an URL that was not traceable using any online search engine throughout the study period; this was to prevent employers of participants in the control group being exposed to the intervention.

#### Intervention group

Participants randomised to the intervention were asked to inform their employer about the online toolbox, either by e-mail with attached secured PDF or by printed letter (both supplied by the research team). The PDF and letter complied fully with privacy and security regulations, and contained information about the participant’s involvement in the study, the URL of the toolbox, and a short promotional video to persuade the employer to visit the toolbox. The employer was asked to watch the video and to use the online toolbox during the participant’s sick leave and RTW. A comparable method to inform employers about the online toolbox, i.e. a one-off provision of the URL of the toolbox, has previously led to a reasonable use of it: 82% of the participating employers used the toolbox, typically 2–3 times over a period of 6 weeks, and on average for 26 min per visit [26].

#### Control group

Participants randomised to the control group were not able to inform their employer about the online toolbox for a period of 6 months. Consequently, they received “care as usual” from their employer. After this follow-up period of 6 months, they were able to inform their employer about the online toolbox, using the same materials as the intervention group.

### Outcomes

Data was collected using the electronic data-capture system Castor [39]. Participants received an invitation to fill out the questionnaires at baseline (T0, before randomisation), and at 3 and 6 months into follow-up (T1 and T2, respectively). In the event that a participant failed to complete 1 of the questionnaires, a reminder was sent by e-mail after 1 week and a telephone call made to them after 2 weeks.

#### Participant characteristics

The T0 questionnaire included questions about the participant’s sociodemographics (i.e. age, gender), health (i.e. diagnosis, treatment, and experience type, based on 3 predefined types [25]) and work (i.e. position, workload, size and sector of the organisation, and duration of sick leave). All questionnaires collected information on the participant’s involvement in any co-interventions, the number of contact moments with their employer since diagnosis (in T0) or in the previous 3 months (in T1 and T2), and their RTW phase.

#### Feasibility of a future definitive RCT

The following method and protocol-related measures were tracked to study feasibility of a future definitive RCT [31, 40]: *reach* (number of potential eligible cancer survivors who received an invitation to participate in the study), *appropriateness of inclusion criteria* (number of potential eligible survivors not able to participate because of each inclusion criteria), *recruitment rate* (percentage of potential eligible survivors included), and *retention rate* (percentage of participants not lost to follow-up). The *appropriateness of the study protocol* was determined on the basis of the percentage of participants who dropped out due to the randomisation procedures or due to randomisation in the control group (*appropriateness of randomisation procedures*), and the percentage of participants who dropped out due to the procedures related to informing their employer, as well as the percentage of participants in the intervention group who did not inform their employer about the online toolbox (*appropriateness of procedures of informing the employer*) [40].

#### Effect measures

Measures for preliminary effect evaluation were collected at each time point (i.e. T0, T1 and T2). The primary effect measure was ‘successful RTW’, which is a combination of 2 components:

1) RTW, which all participants were asked about. Specifically, ‘Have you performed any work activities in the past 4 weeks? This can be either your own original work activities or adapted work activities replacing your original work’ [yes/no].

2) The degree of success of RTW (if returned), measured using the Successful Return-To-Work questionnaire for Cancer Survivors (I-RTW_CS) [41]. This covers 7 items deemed to constitute successful RTW, measuring how the participant perceives their importance and successful accomplishment. Each item’s success score [1–6 rating scale] is weighted by its perceived importance [1–5 rating scale] by multiplying the success and importance scores, which results in that item’s weighted score. The sum of all items’ weighted scores divided by the sum of all their importance scores is the final I-RTW-CS score [range: 1–6]. This ensures that the more important a person perceives an item to be, the larger the contribution it makes to their I-RTW_CS score [41].

Secondary effect measures were:

1) The Quality of Working Life Questionnaire for Cancer Survivors (QWLQ-CS) [42]. This measure is about survivors’ experiences and perceptions in the work environment in the previous 4 weeks, and thus was only completed by the subgroup of participants who did return to work.

2) Unwanted work changes since diagnosis (at T0) and in the previous 3 months (at T1 and T2).

### Randomisation

The electronic data-capture system Castor was used to randomise participants into the intervention or control group [39], with the allocation ratio set at 2:1. This ratio was chosen to include a larger sample in the intervention group and thereby provide more information on the feasibility criteria of the study design. Randomisation was controlled for the participant’s RTW status, i.e. having performed work activities in their own or substitute work during the previous 4 weeks (yes versus no), since it is not possible to evaluate the effect on successful RTW reliably when RTW itself (which is a prerequisite for successful RTW) is unequally distributed over the 2 groups at baseline. Neither the participant nor the research team was blinded for the randomisation.

### Statistical methods

All data was analysed using SPSS 24.0 (IBM, NY, USA). Data entry and analyses were double checked. Data was analysed according to the intention-to-treat principle. All participant characteristics, feasibility data and data for the effect evaluation were assessed using descriptive statistics. *P-*values < 0.05 were considered statistically significant.

#### Feasibility of a future definitive RCT

In the absence of guidelines for assessing feasibility of a future definitive RCT, we formulated the main uncertainties concerning the feasibility of studying the effectiveness of the MiLES intervention in such a future definitive RCT, and specified criteria for each of these uncertainties:

1) Criterion for appropriateness of inclusion criteria and for recruitment: ≥ 70% of the individuals who gave permission for telephone contact are willing to participate in the study;

2) Criterion for appropriateness of protocol: ≤ 20% do not want to participate due to the randomisation procedure or are lost to follow-up due to randomisation in the control group;

3) Criterion for appropriateness protocol: ≤ 20% of the participants are not willing to inform their employer about the online toolbox after randomisation into the intervention group;

4) Criterion for retention rate: ≤ 20% of the participants are lost to follow-up; and,

5) Criterion for reach: the sample size number of 90 participants is included within the recruitment period of 6 months, starting when the first individual is invited by their treating physician.

A future definitive RCT to study the effectiveness of the MiLES intervention was considered feasible when all of these criteria were met. If not, adjustments for the study protocol were formulated.

#### Effect evaluation

Equal distribution between the intervention and control group at baseline was determined using chi-square tests for categorical variables and Student’s t-tests (normally distributed variables) or Mann-Whitney U tests (not normally distributed variables) for continuous variables. When no statistically significant unequal distributions were found at baseline, the relative risk (RR) was determined for RTW at T1 and T2, and a longitudinal multilevel analysis was performed with the group classification as an independent variable and I-RTW_CS scores at the different time point (T0, T1 and T2) as dependent variables.

In the event of significant imbalance of a prognostic factor for the primary effect measure at baseline [13, 43], this factor was used as a covariate for the analysis of that effect measure. In that case, a logistic regression analysis was used to examine differences between the intervention and the control group with regard to RTW. For the subgroup of participants who did return to work, a longitudinal multilevel analysis was performed to examine the difference between both groups with regard to I-RTW_CS scores. We hypothesised that participants in the intervention group 1) returned to work more often and 2) with a higher I-RTW_CS score, compared to the control group.

A longitudinal multilevel analysis was performed for the secondary outcome effect measures. The group classification was set as independent variable and the QWLQ-CS score as dependent variable. For unwanted work changes (i.e. at least 1 unwanted work change), the RR was determined at T1 and T2.

## Results

### Recruitment

**Fig. 1:**
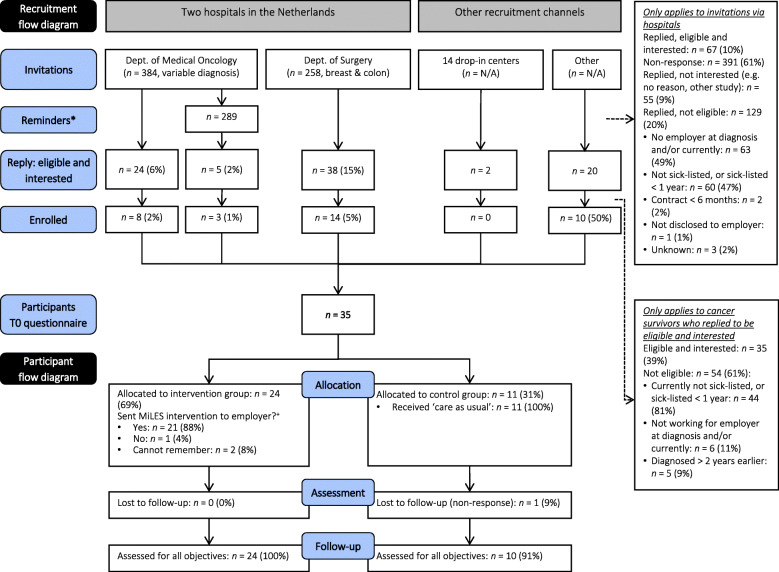
Recruitment and participant flow diagram. N/A = not applicable. *One hospital sent reminders to those who did not respond to the initial invitation within 3 weeks. Some survivors (*n* = 24) were no longer eligible at the time the reminders were sent, as their diagnosis was > 2 years earlier or they were > 63 years of age at that time. ^+^Assessed in T2 questionnaire

Participants were enrolled in the study between April and October 2019, after which enrolment stopped due to time constraints. A total of 642 potential eligible cancer survivors received a personal invitation to participate in the study from a medical specialist in the department where they were being treated, and 289 of them received a reminder. In all, 391 (61%) did not respond to the invitation and, if applicable, the reminder. Another 129 (20%) indicated that were not eligible to participate, and 55 (9%) indicated that they were interested in participating. Sixty-seven (10%) did indicate an interest in participating, but 42 of them were not eligible; mostly because they were currently not sick-listed or had been sick-listed for more than 1 year. Twenty-five survivors – 4% of the initially invited group – were both eligible and interested in participating, and all of them were included in the study. The recruitment flow diagram is shown in Fig. 1.

No cancer survivors were included via the drop-in centres for cancer survivors. Fourteen of these centres were willing to help recruit participants, either by handing out leaflets or through an invitation in their newsletter, but the centres were not being well-used during the summer period and most of their clients were either > 63 years of age or not interested because they felt not ready yet to return to work (although this was not an exclusion criterion). Open invitations via newsletters, social media, and invitations sent by Dutch cancer-related websites recruited 10 participants.

Altogether, 35 cancer survivors were included, and all filled out the baseline questionnaire and were randomised: 24 to the intervention group and 11 to the control group. The response rate for the T1 and T2 questionnaires was 97% (*n* = 34) in both cases. The participant flow diagram is shown in Fig. 1.

### Baseline data

Table 1 summarises the participant characteristics of the intervention and control groups at baseline. Most participants were female (*n* = 34, 97%), were diagnosed with breast cancer (*n* = 28, 80%), and had undergone at least surgery (*n* = 32, 91%) and/or chemotherapy (*n* = 22, 63%). The majority had been working for their current organisation for more than 5 years (*n* = 29, 83%), and 32 (91%) had had more than 3 contact moments with their employer in the period between diagnosis and the T0 questionnaire. At baseline, the participants were in either phase 2 (*n* = 13, 37%), phase 3 (*n* = 10, 29%), or phase 4 (*n* = 12, 34%), and the total duration of their sick leave was 8 ± 3 months at that time. At baseline, the intervention and control groups differed significantly on 1 prognostic factor for RTW [13, 43]: participants randomised to the control group had undergone chemotherapy more often than those randomised to the intervention group (*n* = 10, 91% versus *n* = 12, 50%; *p* = 0.02).

**Table 1:** Participant characteristics at baseline

***Participant characteristics (n = 35)***		***Intervention group*** ***n = 24***		***Control group*** ***n = 11***		***P-value***
		*n (%)*	*Mean ± SD*	*n (%)*	*Mean ± SD*	
**Sociodemographic variables**						
**Age** (years)	18–49	7 (29%)		6 (55%)		0.20
	50–59	13 (54%)		5 (46%)		
	60–63	4 (17%)		0 (0%)		
**Gender**	Female	24 (100%)		10 (91%)		0.14
**Level of education**	Low	1 (4%)		0 (0%)		0.79
	Middle	10 (42%)		5 (46%)		
	High	13 (54%)		6 (55%)		
**Health-related variables**						
**Cancer diagnosis**	Breast	19 (79%)		9 (82%)		0.96
	Colon	2 (8%)		1 (9%)		
	Other (bladder, prostate, ovarian)	3 (13%)		1 (9%)		
**Time since most recent cancer diagnosis**	(months)		10.0 ± 5.5		9.1 ± 3.1	0.61
**Treatment**^**a**^	Surgery	22 (92%)		10 (91%)		0.94
	Chemotherapy	12 (50%)		10 (91%)		**0.02**
	Radiation	11 (46%)		5 (46%)		0.98
	Hormone treatment	9 (38%)		4 (36%)		0.95
	Other (immunotherapy. Oxygen therapy, chemo flush)	3 (13%)		2 (18%)		0.66
**Duration of treatment**	(months)		7.8 ± 4.2		7.9 ± 3.4	0.94
**“Experience type” cancer survivor** [25]	An emotional cancer survivor	0 (0%)		2 (18%)		0.05
	A cancer survivor who wants little attention for his or her health situation	8 (33%)		4 (36%)		
	A cancer survivor who starts looking differently at work and life	13 (54%)		2 (18%)		
	“I cannot judge”	3 (13%)		3 (27%)		
**Work-related variables – job characteristics**						
**Workload**	Physical (0–28)^b^		4.0 ± 3.3		3.1 ± 3.0	0.44
	Emotional (0–28)^b^		6.5 ± 3.7		5.3 ± 3.6	0.38
**Position**	Managerial position	6 (25%)		2 (18%)		0.66
**Gross monthly income**	≤ €2000	3 (13%)		2 (18%)		0.95
	€2001 – €3000	10 (42%)		5 (46%)		
	≥ €3001	8 (33%)		3 (27%)		
	Unknown	3 (13%)		1 (9%)		
**Contract**	Permanent	23 (96%)		10 (91%)		0.56
	Hours		28.9 ± 8.4		29.1 ± 4.5	0.82
**Work-related variables – organisational characteristics**						
**Size of organisation**	< 50 employees	3 (13%)		2 (18%)		0.33
	51–250 employees	4 (17%)		4 (36%)		
	> 251 employee	17 (71%)		5 (46%)		
**Sector**	Non-profit	11 (46%)		5 (46%)		0.98
	Profit	13 (54%)		6 (55%)		
**Time working for current organisation**	≤ 5 years	4 (17%)		2 (18%)		0.84
	6–20 years	11 (46%)		6 (55%)		
	≥ 21 years	9 (38%)		3 (27%)		
**Work-related support since diagnosis**^**a**^	Occupational physician	18 (75%)		9 (82%)		0.66
	Re-integration services	0 (0%)		1 (9%)		0.13
	Social worker	1 (4%)		2 (18%)		0.17
	Supervisor	15 (63%)		7 (64%)		0.95
	Colleagues	1 (4%)		1 (9%)		0.56
	No work-related support	4 (17%)		2 (18%)		0.91
**Contact with employer since diagnosis**	0 times	0 (0%)		0 (0%)		0.27
	1–3 times	2 (8%)		0 (0%)		
	4–9 times	7 (29%)		6 (55%)		
	≥ 10 times	15 (63%)		5 (46%)		
**Work-related variables – current and former work status**						
**Total duration sick leave**	(months)		7.5 ± 3.3		8.4 ± 3.4	0.50
**RTW phase of cancer survivor** [16]	Phase 1: disclosure	0 (0%)		0 (0%)		0.22
	Phase 2: treatment	8 (33%)		5 (46%)		
	Phase 3: RTW planning	9 (38%)		1 (9%)		
	Phase 4: actual RTW	7 (29%)		5 (46%)		
**Current work situation relative to that before cancer diagnosis**^**a**^	Changed working hours	10 (42%)		4 (36%)		0.77
	Changed working tasks	4 (17%)		1 (9%)		0.55
	Assistance at work	0 (0%)		2 (18%)		**0.03**
	Changed workplace	2 (8%)		1 (9%)		0.94
	Nothing has changed	7 (29%)		3 (27%)		0.91
	Not applicable	2 (8%)		2 (18%)		0.15
**Work-related variables – effect measures**						
**Successful RTW**	RTW - Yes	14 (58%)		6 (55%)		0.83
	Success of RTW^c^ (1–6)^b^		5.0 ± 0.7		5.2 ± 0.2	0.49
**Quality of working life**^**c**^	(0–100)^b^		73.9 ± 9.3		72.9 ± 8.6	0.83
**Unwanted work changes**	(relative to pre-diagnosis)		0.0 ± 0.0		0.0 ± 0.0	N/A

^a^Multiple options may apply to 1 participant

^b^Higher scores represent higher levels of workload and of successful RTW

^c^Determined for the subgroup of participants who did return to work

*N/A* not applicable

### Outcomes and estimation

#### Feasibility of a future definitive RCT

Both predefined criteria concerning the study protocol and the criterion concerning the retention rate were achieved (Table 2). The predefined criterion concerning the study’s reach was not achieved; 35 participants were included in 6 months, instead of the target sample size of 90. Lastly, concerning the criterion for the study’s inclusion criteria and recruitment, it appeared that 39% of the individuals who gave permission for telephone contact were actually included. The others gave permission for telephone contact, but turned out not to be eligible. Those actually eligible were all willing to participate and so were all included in the study.

**Table 2:** Results with regard to the predefined criteria for the feasibility of studying the effectiveness of the MiLES intervention in a large-scale RCT, including proposed adjustments to the study protocol

***Feasibility criteria***			
***Category***	***Predefined criteria***	***Result***	***Achieved [yes/no]***
**Inclusion criteria and recruitment**	≥70% of the individuals who gave permission for telephone contact are willing to participate in the study.	Including individuals who eventually were not eligible to participate: 39% Excluding individuals who eventually were not eligible to participate: 100%	Interpretable
**Protocol**	≤20% do not want to participate due to the randomisation procedure or are lost to follow-up due to randomisation in the control group.	0%	Yes
**Protocol**	≤20% of the participants are not willing to inform their employer about the online toolbox, after randomisation into the intervention group	0% were not willing (indicated after randomisation) 4% did not inform their employer (indicated in T2 questionnaire) 8% could not remember whether they had informed their employer (indicated in T2 questionnaire)	Yes
**Retention rate**	≤20% of the participants are lost to follow-up	3%	Yes
**Reach**	90 participants are included within the recruitment period of 6 months, starting when the first individual is invited by his/her treating physician	35	No

*N/A* not applicable

#### Primary and secondary effect measures

The results of the primary and secondary outcome measures are shown in Table 3. The RTW rate at T0, T1, and T2 were 58, 79, and 92%, respectively, for the intervention group and 55, 80, and 100% for the control group (Table 3). The RR of returning to work for the intervention versus the control group at T2 was 0.92 (95% CI: 0.81–1.03). Although participants randomised to the control group had undergone chemotherapy more often than participants randomised to the intervention group (*n* = 10, 91% versus *n* = 12, 50%; *p* = 0.02), we did not adjust any of the analysis since our sample size was too small and chemotherapy was not significantly associated with any of the primary or secondary effect measures in our sample (data not shown). The mean I-RTW_CS score of the individuals who did return to work was 5.0 at all time points for the intervention group (SD ±0.6–0.7), and 5.2 ± 0.2, 5.1 ± 0.2, and 5.0 ± 0.3 for the control group at T0, T1, and T2, respectively. No significant differences were found between the 2 groups in respect of their QWLQ-CS scores over time (*p* = 0.91). The RR of experiencing unwanted work changes for the intervention versus the control group at T2 was 0.42 (95% CI: 0.03–6.03) for the intervention versus the control group.

**Table 3:** Preliminary results for the effectiveness of the MiLES intervention; results of the primary and secondary effect measures over time

		*Group*	***T0 – baseline***		***T1–3 months***		***T2–6 months***		***Statistics***
			*n (%)*	*Mean* ± SD	*n (%)*	*Mean* ± SD	*n (%)*	*Mean* ± SD	
**Primary effect measure**									
**Successful RTW**	RTW – Yes Successfulness of RTW^a^ (1–6)^b^	InterventionControl Intervention Control	14 (58) 6 (55)	5.0 ± 0.7 5.2 ± 0.2	19 (79) 8 (80)	5.0 ± 0.6 5.1 ± 0.2	22 (92) 10 (100)	5.0 ± 0.7 5.0 ± 0.3	RR: .92^c^, 95% CI: 0.81–1.03 *F* = .10^d^, *p* = .91
**Secondary effect measures**									
**Quality of working life**^**a**^	(0–100)^b^	Intervention Control		73.9 ± 9.3 72.9 ± 8.6		73.2 ± 11.2 74.0 ± 7.3		71.4 ± 12.8 73.0 ± 6.1	*F* = .09^d^, *p* = .91
**Unwanted work changes**	(relative to pre-diagnosis)	Intervention Control		0.0 ± 0.0 0.0 ± 0.0		0.1 ± 0.3 0.0 ± 0.0		0.0 ± 0.2 0.1 ± 0.3	RR: .42^c^, 95% CI: 0.03–6.03

^a^Determined for the subgroup of participants who did return to work

^b^Higher scores represent a higher level of successfulness of RTW and quality of working life

^c^Relative risk of returning to work / experiencing unwanted work changes for the intervention versus the control group at T2

^d^Interaction effect between time (T0-T2) and group

#### Sample size future definitive RCT

A sample size calculation for a future definitive RCT on the effectiveness of the MiLES intervention was not considered relevant given the outcomes for the primary effect measure “successful RCT”.

## Discussion

This randomised feasibility trial aimed to assess feasibility of a future definitive RCT on the effectiveness of the MiLES intervention targeted at employers, for successful RTW of cancer survivors. Most predefined criteria for feasibility of such a future definitive RCT were met, e.g. retention rates were minimal and the study protocol was found to be acceptable. However, the predefined criterion concerning the study’s reach was not met, i.e. 35 instead of 90 cancer survivors were included, and the study’s design has led to a considerable selection bias towards cancer survivors at low risk of adverse work outcomes. A future definitive trial on the effectiveness of the MiLES intervention should therefore apply an alternative design. Preliminary results for the primary and secondary effect measures showed no difference between the intervention and control group over time, which did not allow a sound sample size calculation for a future definitive RCT on the effectiveness of the MiLES intervention.

### Strengths and limitations

A strength of the current randomised feasibility trial is that several process measures were monitored and reported, such as the efficacy of different recruitment strategies, the reasons for cancer survivors not to participate in the study, and the retention of participants throughout the different steps of the protocol. This has resulted in a comprehensive insight into the feasibility of a future definitive RCT and uncovered the main bottlenecks regarding the execution of such a trial, and thereby provides the opportunity to adjust the design with these bottlenecks taken into consideration. This can also potentially obviate resource-intensive and unfeasible studies [31]. Another strength of this study is the use of the combined primary outcome measure “successful RTW”. This allows a broader and more meaningful evaluation of a cancer survivor’s RTW [41], since we do not assume that the return to work itself is necessarily always the desired end point for the cancer survivor. Instead, the individual desired characteristics of the RTW also count [41, 44]. Lastly, the current study design enabled us to intervene on employers and concomitantly to measure work outcomes of cancer survivors, while respecting all ethical and privacy-related concerns.

A limitation of the current study is that only 4% of the group initially approached was included, due to non-response (61%), non-eligibility (26%), or no interest (9%). Note that it is unclear whether the non-responders were eligible to participate, since their employment status was unknown at the hospitals that sent the invitations. Those included, moreover, were a homogeneous, very select group: female breast-cancer survivors with a permanent employment contract, working in a job with low physical strain, and who – looking at the years they had worked for their current organisation and the large number of contact moments with their employer before enrolling in the study – can reasonable be assumed to have a relatively good relationship with their employer. Although this is an important outcome of this randomised feasibility trial, it also affects the generalisability of its results, i.e. the outcomes on feasibility of a future definitive RCT and the preliminary results for the effect measures. Another limitation is that survivors on average enrolled almost 1 year post-diagnosis, and therefore already had a relatively lengthy cancer-related dialogue with their employer. Since satisfactory work-related conversations at an early stage are recommended [16, 19, 45], enrolling at later stages may reduce the effects of the intervention. The lack of data on exposure at the employer level can also be regarded a limitation. Privacy regulations and ethical concerns did not allow the measurement of both employers’ user data concerning the intervention and work outcomes of the cancer survivors in the same study, which in turn precluded the conduct of per-protocol analyses. However, our study on the use of the online toolbox has provided some substantiation that the method used to inform employers about the toolbox, i.e. a one-off provision of URL of the toolbox, actually leads to use among employers [26]. An additional process evaluation parallel to the current randomised feasibility study, e.g. interviews with participants in the intervention group, could have offered some more insight into the transfer of the intervention to the employer as well as delivering a rough estimate of the “dose delivered”. Lastly, the outcomes for the primary effect measure “successful RTW” did not allow a sound sample size calculation for a future definitive RCT on the effectiveness of the MiLES intervention, which was stated initially as a secondary aim of the current study.

### Implications of the findings

In the current study, 4 out of 5 predefined criteria for feasibility of a future definitive RCT on the effectiveness of the MiLES intervention were met. Only the recruitment of cancer survivors fell short of the predefined criterion. Theoretically, therefore, we could intensify the recruitment strategies for the future definitive RCT to comply with the latter criterion as well; for example, by involving more hospitals or increasing publicity via social media. However, only 4% of the potential eligible participants who received an invitation via their treating physician ultimately participated, which is substantially lower than the rate for other cancer-related studies with comparable recruitment strategies, i.e. 37% [46], and around 20–25% [47]. Reasons for the low inclusion rate are that cancer survivors were invited without knowing their eligibility, e.g. their current work status, and, hypothetically, their reservations about participating in a study on the effectiveness of an intervention targeted at their employer. In addition, the drop-in centres mentioned that survivors were not interested in participating because they felt not yet ready to return to work, although this was not utilised as an inclusion criterion for the study. These issues during recruitment led to the identified selection bias, which in turn affected the generalisability of the outcomes to a broad group of cancer survivors. The group that did participate was highly homogeneous in terms of gender and diagnosis, and rated the level of success of their RTW and the quality of their working life relatively high at baseline. That is, their quality of working life at baseline, e.g. on average 10 months after diagnosis, was very similar to that found in a previous study with cancer survivor who had been diagnosed 0–10 years earlier (QWLQ-CS score of 74 ± 9 versus 75 ± 12, respectively) [47]. As a result of the homogeneous sample, both the preliminary results on the effectiveness of the MiLES intervention on successful RTW of cancer survivors and the result on feasibility of a future definitive RCT should be interpreted with caution. Intensifying recruitment strategies alone would not, we can assume, solve the issues regarding selection bias and the generalisability of the outcomes.

### Methodological considerations for the study of future employer-based interventions

Findings from the current randomised feasibility trial can provide valuable input for the development of a future definitive trial on the effectiveness of employer-based interventions on work outcomes of cancer survivors. The main challenges lie in providing the employer with an intervention of interest, and in embedding this in a study design that allows the measurement of work outcomes for the cancer survivor in their employ. In the current randomised feasibility trial, the design was initiated via the cancer survivor. This has some advantages, such as 1) fully respecting survivors’ privacy, 2) enabling us to invite large numbers of survivors at the same time via medical specialists, and 3) letting survivors themselves decide whether or not to inform their employer about the MiLES intervention [48]. But some drawbacks have also been identified, e.g. relatively low inclusion rates and selection bias towards cancer survivors at low risk of adverse work outcomes. As a result, adjustments to the study protocol are needed, mainly with regard to the recruitment strategies employed. Face-to-face invitations soon after diagnosis, e.g. by medical specialists, specialised oncological nurses, or occupational physicians, may enhance the participation rate, reduce selection bias, and better target cancer survivors at risk of adverse work outcomes as also recommended by previous studies [9, 49] and those whose employer may benefit from an employer-based intervention. However, the workload in the clinical setting is high, which may hinder recruitment [50, 51]. Alternatively, consistent documentation of cancer survivors’ employment status in their electronic patient record would be an important first step, since this enables better targeting of eligible survivors, reduces the recruitment efforts required, and prevents the unnecessary burdening of non-eligible survivors.

As an alternative, the study could also be initiated via the employer. But a design in which cancer survivors are recruited directly by their employers is prohibited under privacy regulations [29], which not allow employers to process health-related information about their employees. In order to comply with those regulations, this alternative is thus only permissible when the intervention is implemented organisation-wide, followed by recruitment of cancer survivors working for that organisation for a questionnaire study; for example, by their occupational physician [29, 52, 53]. Such a design may also limit selection bias, since cancer survivors are not asked to participate in an intervention study in which they are asked to inform their employer about the intervention. Possible drawbacks are the strong dependence on the effectiveness of the implementation plan – since an effective one can be regarded as a prerequisite for the employer being exposed to the intervention – [52], difficulties in implementing a control group, and potential difficulties in recruiting sufficient cancer survivors since the incidence of the disease per organisation is, fortunately, low.

### Recommendations for further research

The abovementioned methodological considerations with regard to a future definitive design to study the effectiveness of employer-based interventions on work outcomes of cancer survivors clearly demonstrate the complexity of studying such an intervention, with a multitude of factors and stakeholders involved [54, 55]. On the basis of the results in the current randomised feasibility trial, we do not recommend proceeding with a future definitive RCT using the current design, since we consider this inappropriate to gather reliable outcomes on the effectiveness of the MiLES intervention on successful RTW of cancer survivors. Instead, we recommend involving all relevant stakeholders in the development of an adequate study design to determine the effectiveness of the MiLES intervention. Involvement of stakeholders in the development of interventions (e.g. [22]) and in the development of protocols to evaluate these interventions (e.g. [56]) has already been recommended and frequently employed [49, 57]. Stakeholder involvement in the development of study designs to evaluate interventions is less common, yet recommended or even a prerequisite from subsidising bodies [58]. Such involvement, e.g. by organising focus groups with cancer survivors, employers, and occupational and treating physicians, may provide an interesting opportunity to determine an adequate and feasible study design for the evaluation of complex interventions, such as MiLES. This should lead to a study design that aligns with all privacy regulations and stakeholder preferences, is feasible in actual practice, and has adequate internal and external validity.

Secondly, we noticed a lack of agreement on the use of the terms “feasibility” and “pilot” in international literature [33, 35]. We here regard feasibility as the overarching concept for studies assessing whether a future definitive trial can be done [32]. For studies conducted in preparation for an RCT assessing the effect of an intervention, 3 distinct study types are distinguished under the umbrella of feasibility studies [32]: 1) randomised pilot studies (also called “randomised feasibility studies”), which reflect the future definitive trial on most parts, including randomisation; 2) non-randomised pilot studies, which are similar to randomised pilot studies, but without randomisation of participants; and 3) feasibility studies that are not pilot studies, which are aimed to study whether an element of the future trial can be done (i.e. the intervention itself or other processes of the future trial are not implemented in these studies). The main difference between these study types is thus the degree to which the feasibility study reflect the design of the future definitive trial. To avoid misconceptions, we recommend future research to regard feasibility as an overarching concept, and to consistently use the above-mentioned terminology to refer to the different types of feasibility studies [32]. Thirdly, the predefined criteria for feasibility of a future definitive RCT formulated for the current randomised feasibility trial were all related to its process, i.e. recruitment rates, retention rates, and acceptability of the study protocol [31]. Although most of these criteria were met, we can assume that, due to the select group of cancer survivors included, a future definitive RCT would not result in outcomes with high external generalisability. We therefore recommend that future randomised feasibility trials not only formulate criteria for the number of participants included, but also supplement these with criteria concerning the heterogeneity of those participants, e.g. regarding their gender, diagnosis, or level of education. This could enhance the validity of the outcomes of the future definitive trial.

## Conclusions

The study protocol employed in the current randomised feasibility trial turned out to be acceptable, and retention rates were minimal. However, recruitment of cancer survivors fell short of the predefined minimum for feasibility of a future definitive RCT using the current study design and, more importantly, the study design resulted in a considerable selection bias towards cancer survivors at low risk of adverse work outcomes. The outcomes on feasibility of a future definitive RCT and on the preliminary effect measures may therefore not be generalisable to a broad group of cancer survivors. The effectiveness of the MiLES intervention should therefore be investigated using an alternative study design, and relevant stakeholders should be involved in the development of that design. The findings from the current randomised feasibility trial provide valuable input for future designs to study the effectiveness of employer-based interventions on work outcomes of cancer survivors, as well as for the feasibility of such studies and the generalisability of their outcomes.

## Supplementary Information

**Additional file 1.** Completed CONSORT checklist (extension for randomised pilot and feasibility trials)

**Additional file 2.** Study protocol of ‘A randomised feasibility trial of an employer-based intervention for enhancing successful return to work of cancer survivors (MiLES Intervention)’

## Data Availability

The datasets used and/or analysed during the current study are available from the corresponding author on reasonable request.

## Abbreviations

CONSORT: Consolidated Standards of Reporting Trials
I-RTW_CS: Successful Return-To-Work questionnaire for Cancer Survivors
MiLES: The Missing Link: optimising return to work of Employees diagnosed with cancer, by Supporting employers.
N/A: Not applicable
NTR: Dutch Trial Register (Dutch: Nederlands Trial Register)
QWLQ-CS: Quality of Working Life Questionnaire for Cancer Survivors
RCT: Randomised controlled trial
RR: Relative risk
RTW: Return to work
SD: Standard deviation
