# Supplementary material for: A randomised feasibility trial of an employer-based intervention for enhancing successful return to work of cancer survivors (MiLES intervention)
Source: BMC Public Health. 2021 Jul 21;21:1433. doi: 10.1186/s12889-021-11357-9 (PMC8293550; doi:10.1186/s12889-021-11357-9)
Supplement: Supplementary file 2 — Additional file 2. Study protocol of ‘A randomised feasibility trial of an employer-based intervention for enhancing successful return to work of cancer survivors (MiLES Intervention)’ [file 12889_2021_11357_MOESM2_ESM.doc]

# Additional file 2. Study protocol of ‘A randomised feasibility trial of an employer-based intervention for enhancing successful return to work of cancer survivors (MiLES Intervention)’

## Research protocol: part 1

### Project summary

The study will be conducted as a randomized feasibility trial with a follow up of 6 months. Participants will be employees diagnosed with cancer <2 years earlier, 18-63 years, currently in paid employment, and fully or partially sick-listed <1 year. Participants will be randomised to an intervention or a waiting list control group. The intervention itself targets the employer (i.e. supervisor, HR-manager or case-manager) of the included employee with cancer. The intervention can be seen as a ‘web-based intervention’. This web-based intervention, an online toolbox, is an open access website consisting of supportive information on how to guide employees with cancer throughout different return to work (RTW) phases. This supportive information consists of to-the-point text and video’s, and targets several actions of the employer, e.g. communicating effectively with the employee with cancer, assessing the employee’s work ability and supporting the employee with cancer emotionally. This study aims to assess feasibility of a future definitive randomised controlled trial (RCT) on the effectiveness of the MiLES intervention. Feasibility of a future definitive RCT was determined on the basis of predefined criteria related to method and protocol-related uncertainties (e.g. reach, retention, appropriateness). Also preliminary results on the effectiveness of the MiLES intervention will be obtained and the sample size needed in a future definitive RCT on the effectiveness of the MiLES intervention will be determined. The online toolbox will be considered effective if the intervention group improved significantly more on both components of the primary outcome measure ‘successful RTW’: the number of participants in the intervention group that return to work is higher compared with the control group, provided that their degrees of successfulness of RTW is also significantly higher. Data will be collected from 2019-01-01 to 2020-01-01. We expect a future definitive RCT to be feasible.

### General information

Protocol title: The missing link: optimizing return-to-work for employees with cancer, by supporting employers

Protocol identifying number: Trial NL6758 (NTR7627), , dd. 30/10/2018.

Sponsor: Dutch Cancer Society (Delflandlaan 17, 1062 EA Amsterdam, The Netherlands), grant number UVA 2014–7153.

Investigators:

- M. A. Greidanus, MSc:
  - Research site: Department Public and Occupational Health/ Coronel Institute of Occupational Health, Amsterdam Public Health research institute, Amsterdam UMC, University of Amsterdam, The Netherlands
  - Address: Meibergdreef 9, 1100 DD Amsterdam, The Netherlands
  - Responsibilities: Study concept and design, recruitment participants, data collection, data analysis, interpretation data, draft manuscript, and read and approve the final manuscript.
- Prof. A. E. de Rijk, PhD:
  - Research site: Department of Social Medicine, Care and Public Health Research Institute (CAPHRI), Faculty of Health, Medicine and Life Sciences, Maastricht University, Maastricht, The Netherlands.
  - Address: Universiteitsingel 40, 6200 ER Maastricht, The Netherlands
  - Responsibilities: Obtained funding, study concept and design, interpretation data, critically revise manuscript, and read and approve the final manuscript.
- A. G. E. M. de Boer, PhD:
  - Research site: Department Public and Occupational Health/ Coronel Institute of Occupational Health, Amsterdam Public Health research institute, Amsterdam UMC, University of Amsterdam, The Netherlands
  - Address: Meibergdreef 9, 1100 DD Amsterdam, The Netherlands
  - Responsibilities: Study concept and design, interpretation data, critically revise manuscript, and read and approve the final manuscript.
- M. E. M. M. Bos, PhD:
  - Research site: Department of Medical Oncology, Erasmus Medical Center, Rotterdam, The Netherlands
  - Address: Doctor Molewaterplein 40, 3015 GD Rotterdam, The Netherlands
  - Responsibilities: Recruitment participants, critically revise manuscript, and read and approve the final manuscript.
- P. W. Plaisier, PhD:
  - Research site: Department of Surgery, Albert Schweitzer Hospital, Dordrecht, The Netherlands.
  - Address: Albert Schweitzerplaats 25, 3318 AT Dordrecht, The Netherlands
  - Responsibilities: Recruitment participants, critically revise manuscript, and read and approve the final manuscript.
- R. M. Smeenk, PhD:
  - Research site: Department of Surgery, Albert Schweitzer Hospital, Dordrecht, The Netherlands.
  - Address: Albert Schweitzerplaats 25, 3318 AT Dordrecht, The Netherlands
  - Responsibilities: Recruitment participants, critically revise manuscript, and read and approve the final manuscript.
- Prof. M. H. W. Frings-Dresen, PhD:
  - Research site: Department Public and Occupational Health/ Coronel Institute of Occupational Health, Amsterdam Public Health research institute, Amsterdam UMC, University of Amsterdam, The Netherlands
  - Address: Meibergdreef 9, 1100 DD Amsterdam, The Netherlands
  - Responsibilities: Obtained funding, study concept and design, interpretation data, critically revise manuscript, and read and approve the final manuscript.
- S. J. Tamminga, PhD:
  - Research site: Department Public and Occupational Health/ Coronel Institute of Occupational Health, Amsterdam Public Health research institute, Amsterdam UMC, University of Amsterdam, The Netherlands
  - Address: Meibergdreef 9, 1100 DD Amsterdam, The Netherlands
  - Responsibilities: Obtained funding, study concept and design, check data analysis, interpretation data, critically revise manuscript, and read and approve the final manuscript.

### Rationale & background information

We have developed an employer-based intervention, named MiLES (an abbreviation of “the Missing Link: optimizing return to work of Employees diagnosed with cancer, by Supporting employers”) (1). By providing employers with support so as to improve their RTW guidance to cancer survivors, the MiLES intervention aims to enhance successful RTW of cancer survivors (1).

Tools that intervene at the intersection between employer and cancer survivor are scarce (2, 3), and studies to determine the effectiveness of such tools on survivors’ work outcomes are, to the best of our knowledge, absent in scientific literature (2, 3, 4). Several complicating aspects and uncertainties make it is essential to conduct a feasibility trial before conducting a future definitive trial on the effectiveness of the MiLES intervention on successful RTW of cancer survivors (5, 6).

The aim of this randomised feasibility trial is to assess feasibility of a future definitive RCT on the effectiveness of the MiLES intervention in terms of recruitment, reach, and acceptability of the study protocol. Secondary aims are: 1) to obtain preliminary results on the effectiveness of the MiLES intervention on successful RTW of cancer survivors, and 2) to determine the sample size needed in a future definitive RCT on the effectiveness of the MiLES intervention.

### References (of literature cited in preceding sections)

1 Greidanus MA, de Boer A, Tiedtke CM, Frings-Dresen MHW, de Rijk AE, Tamminga SJ. Supporting employers to enhance the return to work of cancer survivors: development of a web-based intervention (MiLES intervention). J Cancer Surviv. 2020;14:200-10.

2. Tikka C, Verbeek JH, Tamminga SJ, Leensen MCJ, De Boer AGEM. Rehabilitation and return to work after cancer: literature review. Luxembourg: European Agency for Safety and Health at Work; 2017.

3. European Agency for Safety and Health at Work. Rehabilitation and return to work after cancer — instruments and practices. Luxembourg: European Union; 2018.

4. Tamminga SJ, Wolvers MDJ, Greidanus MA, Zaman AGNM, Braspenning AM, Frings-Dresen MHW, et al. Employees Diagnosed with Cancer: Current Perspectives and Future Directions from an Employer’s Point of View. J Occup Rehabil. 2018;29(2):472-4.

5. Blatch-Jones AJ, Pek W, Kirkpatrick E, Ashton-Key M. Role of feasibility and pilot studies in randomised controlled trials: a cross-sectional study. BMJ Open. 2018;8(9):e022233.

6. Thabane L, Ma J, Chu R, Cheng J, Ismaila A, Rios LP, et al. A tutorial on pilot studies: the what, why and how. BMC Med Res Methodol. 2010;10:1.

### Study goals and objectives

The aim of this randomised feasibility trial will be to assess feasibility of a future definitive RCT on the effectiveness of the MiLES intervention in terms of recruitment, reach, and acceptability of the study protocol. Secondary, the study will also aim to evaluate the effectiveness of the online toolbox targeting employers on successful RTW of employees with cancer and to determine the sample size needed in a future definitive RCT on the effectiveness of the MiLES intervention. The online toolbox will be considered effective if the intervention group improved significantly more on both components of the primary outcome measure ‘successful RTW’: the number of participant in the intervention group that have returned to work is higher compared with the control group, provided that their degrees of successfulness of RTW is also significantly higher.

### Study design

Type of study: randomized feasibility trial

Inclusion criteria research population: diagnosed with cancer <2 years ago, able to fill out three questionnaires in the next six months, currently (partial) sick-listed for <1 year, of the working age (18-63 years), and in paid employment under a temporary (>6 months remaining) or permanent contract on part-time, full-time of flexible basis.

Exclusion criteria: not yet disclosed being diagnosed with cancer to the employer.

Expected duration study: one year (2019-01-01 until 2020-01-01).

### Methodology

Interventions: the intervention consists of an online toolbox targeting the participant’s employer. With ‘employer’ we refer to the person who is, from his/her role as (direct) supervisor, human resource manager or case-manager, in direct contact with the participant and from there responsible for guiding the participant at work. The online toolbox is an open-assess website containing to-the-point information, videos and checklists to support employers during the RTW process of an employee diagnosed with cancer.

Intervention group: participants randomised in the intervention group will be asked to inform their employer about the online toolbox, either by email or letter. Employers will be asked to use the online toolbox throughout the RTW process of the participant.

Control group: participants randomised to the control group will not inform their employer about the online toolbox for a period of six months.

Primary outcome measure: feasibility of a future definitive RCT on the effectiveness of the MiLES intervention (in terms of reach, appropriateness of inclusion criteria, recruitment rate and appropriateness of the study protocol).

Primary effect measure: the primary effect measure of the intervention is the combined effect measure successful RTW. This effect measure is a combination of RTW (having performed work activities in own work or in replacement work during the past four weeks) and a subgroup analyses for the ones that did return to work: degrees of successfulness of RTW (a new weighted, on individual level, outcome measure) (7). The degree of successfulness of RTW contains seven items, which were found to constitute successful RTW most importantly, according to employees diagnosed with cancer: ‘enjoyment in work’, ‘work without sacrificing health’, ‘open communication with employer’, ‘perceived confidence from employer without assumptions about workability’, ‘feeling welcome at work’, ‘good work-life balance’ and ‘joint satisfaction about the work situation (employer and employee)’. Each participant rates each items on its perceived importance and on its success. The combination of the items’ relative perceived importance and the items’ success determines the degrees of successfulness of RTW.

Secondary effect measures: Quality of Working life (QWLQ_CS) (8) and number of unwanted work changes (#).

Time points data collection: baseline, before randomisation [T0], after 3 months of follow up [T1] and after six months of follow up [T2].

Randomization: using the electronic data-capture system Castor; allocation ratio will be set at 2:1 (intervention vs. control group); controlled the participant’s RTW status; neither the participant nor the research team will be blinded for the randomization.

Graphic outline: see figure 1.


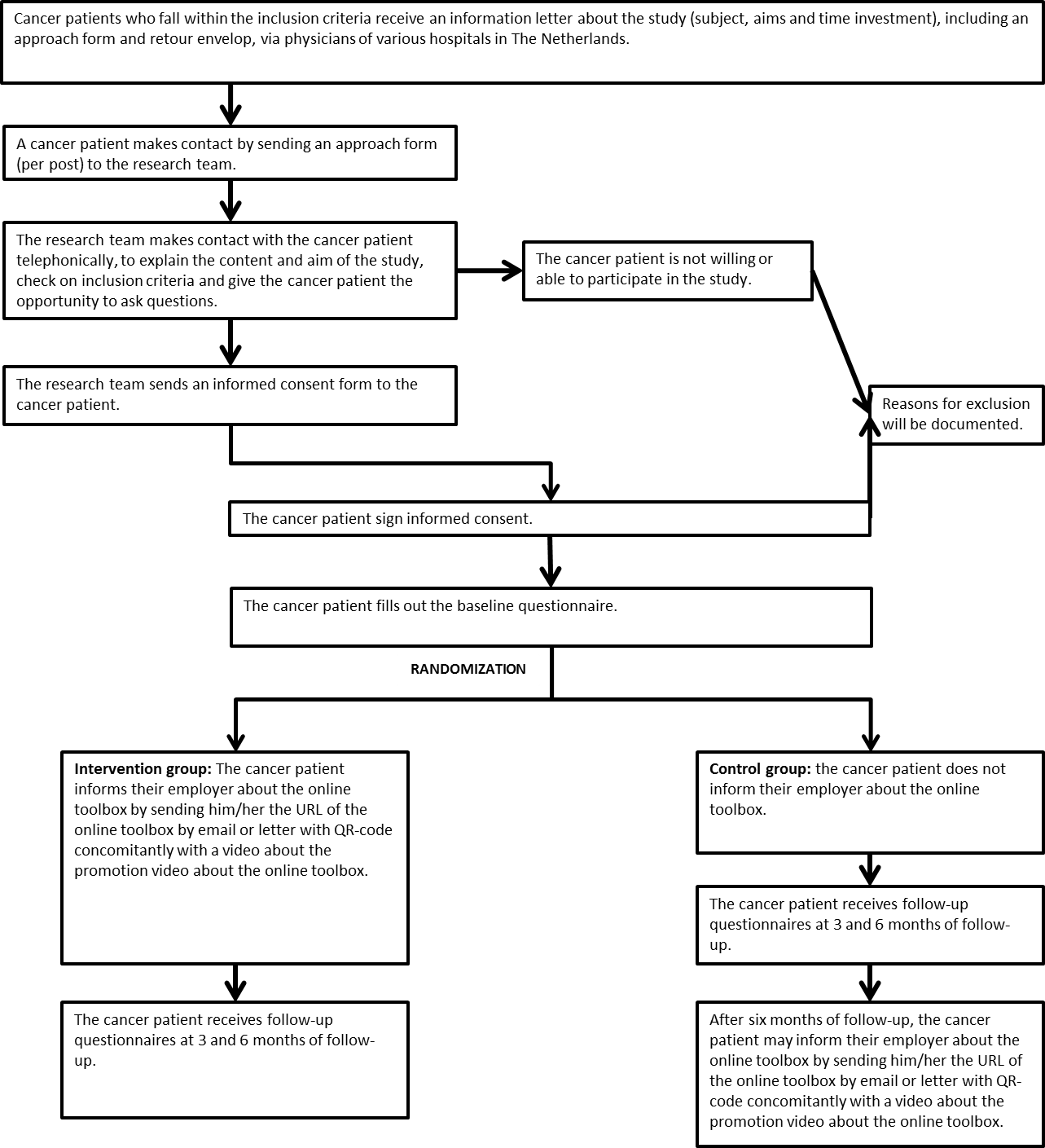


**Figure 1.** Graphic outline of the study design and procedures using a flow diagram.

### Follow-up

Data collection and total follow-up will be six months.

### Data management and statistical analysis

Feasibility: a future definitive RCT to study the effectiveness of the MiLES intervention will be considered feasible when all of following criteria are met:

1. Criterion for appropriateness of inclusion criteria and for recruitment: ≥ 70% of the individuals who gave permission for telephone contact are willing to participate in the study;
2. Criterion for appropriateness of protocol: ≤ 20% do not want to participate due to the randomization procedure or are lost to follow-up due to randomization in the control group;
3. Criterion for appropriateness protocol: ≤ 20% of the participants are not willing to inform their employer about the online toolbox after randomization into the intervention group;
4. Criterion for retention rate: ≤ 20% of the participants are lost to follow-up; and,
5. Criterion for reach: the sample size number of 90 participants is included within the recruitment period of six months, starting when the first individual is invited by their treating physician.

Effect measures: equal distribution between the intervention and control group at baseline will be determined using chi-square tests for categorical variables and Student’s t-tests (normally distributed variables) or Mann-Whitney U tests (not normally distributed variables) for continuous variables. When no statistically significant unequal distributions will be found at baseline, the relative risk (RR) will be determined for RTW at T1 and T2, and a longitudinal multilevel analysis will be performed with the group classification as an independent variable and I-RTW_CS scores at the different time point (T0, T1 and T2) as dependent variables.

In the event of significant imbalance of a prognostic factor for the primary effect measure at baseline (9, 10), this factor will be used as a covariate for the analysis of that effect measure. In that case, a logistic regression analysis will be used to examine differences between the intervention and the control group with regard to RTW. For the subgroup of participants who did return to work, a longitudinal multilevel analysis will be performed to examine the difference between both groups with regard to I-RTW_CS scores. We hypothesised that participants in the intervention group 1) returned to work more often and 2) with a higher I-RTW_CS score, compared to the control group.

A longitudinal multilevel analysis will be performed for the secondary outcome effect measures. The group classification will be set as independent variable and the QWLQ-CS score as dependent variable. For unwanted work changes (i.e. at least one unwanted work change), the RR will be determined at T1 and T2.

### Expected outcomes of the study

The study will inform researchers, policy makers and practitioners in the field of occupation health.

### Dissemination of results and publication policy

The results will be disseminated via an article in a peer-reviewed journal related to public health, presentations at several (intern)national conferences and meetings with health care professionals, researchers, employers and policy makers. The scientific journal in which the article will be published will be acknowledged in all these presentations, as well as the sponsor (Dutch Cancer Society), the Dutch online cancer platform ‘kanker.nl’ for its help in recruiting study participants and research assistant Sonja Brouwers.

### Duration of the project

Development study protocol & ethics approval: 2018/08/01 - 2019/01/01

Participant recruitment: 2019/0101 - 2019/07/01

Data collection: 2019/01/01 - 2020/01/01

Data analysis: 2020/01/01 – 2020/04/01

Writing manuscript: 2020/04/01 – 2021/01/01

### Project management

Executive researcher: M. A. Greidanus, MSc.

Principle investigator: A. G. E. M. de Boer, PhD.

Project leaders: Prof. A. E. de Rijk, PhD, A. G. E. M. de Boer, PhD, Prof. M. H. W. Frings-Dresen, PhD, and S. J. Tamminga, PhD.

### Ethics

The Executive Board of the Medical Ethics Committee of the Amsterdam UMC, location Academic Medical Center (AMC) will decide whether an extensive test of ethical and juridical aspects is necessary (W18_355 # 18.405, dd. 13/11/2018). We expect that this is not necessary, since the burden for participants will be low and their psychological integrity will not be at stake. Nevertheless, all procedures will be in accordance with the ethical standards of the responsible committees on human experimentation (institutional and national) and with the 1964 Helsinki Declaration and its later amendments, or comparable ethical standards. Before they will be included in the study, informed consent will be obtained from all participants by the executive researcher M. A. Greidanus (see Figure 1 for the procedure).

### Informed consent forms

The informed consent form in Dutch (original language):

*Toestemmingsformulier*

voor deelname aan het onderzoek ‘effectiviteit website’ t.b.v. het MiLES project.

Ik verklaar hierbij op duidelijke wijze te zijn geïnformeerd over de aard, doel, risico’s en belasting van het onderzoek en het invullen van de vragenlijsten. Ik ben voldoende in de gelegenheid geweest vragen te stellen over het onderzoek en mijn vragen zijn naar tevredenheid beantwoord. Ook had ik genoeg tijd om te beslissen om mee te doen.

Hierbij verleen ik vrijwillig toestemming voor deelname aan het onderzoek ‘effectiviteit website’ t.b.v. het MiLES project, waarbij onderzoeksgegevens enkel voor onderzoeksdoeleinden gebruikt worden en onderzoeksgegevens 15 jaar na afloop van dit onderzoek bewaard worden. Het doel van het onderzoek is om te onderzoeken of een website voor werkgevers succesvolle werkhervatting van werknemers met een diagnose kanker bevordert.

Alle informatie die door het Coronel Instituut voor Arbeid en Gezondheid wordt verzameld, wordt vertrouwelijk behandeld. Alle onderzoeksgegevens worden niet aan derden verstrekt en zijn alleen in te zien door het onderzoeksteam. Persoonsgegevens zijn niet te herleiden naar mij als persoon en worden nooit gebruikt in studiedocumentatie, rapporten, publicaties of voor verzekeringsdoeleinden.

Ik ben op de hoogte van het recht op inzage in data voortvloeiend uit mijn deelname. Ik ben op de hoogte dat ik mijn antwoorden kan wijzigen en dat ik het recht heb om vergeten te worden (alle data voortvloeiend uit uw deelname wordt door de onderzoeker vernietigd). Ook kan ik op verzoek mijn onderzoeksgegevens over laten dragen en heb ik recht op informatie over het onderzoek en het gebruik van mijn onderzoeksgegevens.

Ik heb te allen tijde, de vrijheid om op deze beslissing terug te komen, zonder dat ik daarvoor een verklaring hoef te geven.

 Ik wil mee doen aan dit onderzoek.

Naam deelnemer: .................................................................................................................

Plaats: datum:........../........../20.........

The informed consent form translated to English:

Informed consent form

for participation in the study "effectiveness of website" for the MiLES project.

I hereby declare that I have been clearly informed about the nature, purpose, risks and burden of the research and the completion of the questionnaires. I have had sufficient opportunity to ask questions about the study and my questions have been answered satisfactorily. I also had enough time to decide about my participation.

I hereby voluntarily consent to participate in the study "effectiveness of the website" for the MiLES project, in which research data is only used for research purposes and research data is stored for 15 years after completion of the study. The aim of the study is to investigate whether a website for employers promotes the successful return to work of employees with a cancer diagnosis.

All information collected by the Coronel Institute for Work and Health is treated confidentially. No research data is provided to third parties and the data can only be viewed by the research team. Personal data cannot be traced back to me as a person and is never used in study documentation, reports, publications or for insurance purposes.

I am aware of the right of access to data arising from my participation. I am aware that I can change my answers and that I have the right to be forgotten (all data resulting from your participation will be destroyed by the researcher). I can also have my research data transferred on request and I am entitled to information about the research and the use of my research data.

I have the freedom to reconsider this decision at any time, without having to provide an explanation.

 I do want to participate in the study.

Name participant: .................................................................................................................

Place: date:........../........../20.........

### References (of literature cited in preceding sections)

7. Greidanus MA, de Boer AGEM, de Rijk AE, Brouwers S, de Reijke TM, Kersten MJ, et al. The Successful Return-To-Work Questionnaire for Cancer Survivors (I-RTW_CS): Development, Validity and Reproducibility. The Patient - Patient-Centered Outcomes Research. 2020;13(5):567–82.

8. de Jong M, Tamminga SJ, de Boer AG, Frings-Dresen MH. Quality of working life of cancer survivors: development of a cancer-specific questionnaire. J Cancer Surviv. 2016;10(2):394-405.

9. Islam T, Dahlui M, Majid HA, Nahar AM, Mohd Taib NA, Su TT, et al. Factors associated with return to work of breast cancer survivors: a systematic review. Bmc Public Health. 2014;14 Suppl 3:S8.

10. van Muijen P, Weevers NL, Snels IA, Duijts SF, Bruinvels DJ, Schellart AJ, et al. Predictors of return to work and employment in cancer survivors: a systematic review. Eur J Cancer Care (Engl). 2013;22(2):144-60.

## Research protocol: part 2

### Budget

Details available upon reasonable request.

### Other support for the project

N.A.

### Links to other projects

The study is part of the research project ‘The missing link: optimizing return-to-work for employees with cancer, by supporting employers’.

### Curriculum Vitae of investigators

Available upon reasonable request.
